# Accuracy of Visual Inspection of Thickened Liquids: Implications for Multidisciplinary Dysphagia Management and Clinical Nutrition

**DOI:** 10.1111/jhn.70294

**Published:** 2026-06-18

**Authors:** Raymond Fong, Connie C. Y. Kwan

**Affiliations:** ^1^ Department of Otorhinolaryngology, Head and Neck Surgery, Faculty of Medicine The Chinese University of Hong Kong Hong Kong SAR China; ^2^ Institute of Human Communicative Research The Chinese University of Hong Kong Hong Kong SAR China

**Keywords:** dysphagia, texture modification, thickened liquid

## Abstract

**Introduction:**

To evaluate the accuracy of visually estimating thickened liquid volume via video‐observed conditions and to determine whether clinical experience or thickener composition influences judgement reliability.

**Methods:**

A cross‐sectional online survey was administered to 57 practising speech‐language pathologists (SLPs) and 50 postgraduate SLP students. Participants viewed four 12‐s videos of fluids prepared to explicit International Dysphagia Diet Standardisation Initiative consistencies (Levels 1, 2 and 4) using starch and xanthan gum thickeners. Participants estimated the volumetric amount of thickener (in product‐specific scoops) added to each sample.

**Results:**

Overall accuracy was low, with no significant difference between students (49.5%) and practitioners (47.4%) (*p* = 0.60). Combined cohort analysis showed that years of clinical practice alone were not associated with improved fluid estimation accuracy (*p* = 0.38). Students significantly outperformed practitioners in identifying Level 4 starch‐based liquids (88.0% vs. 68.4%, *p* = 0.028) but were inaccurate in identifying Level 4 xanthan gum‐based liquids (2.0% vs. 29.8%, *p* < 0.001).

**Conclusion:**

Visual fluid estimation via video is highly variable, and clinical experience alone was not associated with improved accuracy in this cohort. To optimise patient safety and prevent secondary complications like dehydration from over‐thickening, multidisciplinary teams should supplement subjective clinical judgement with objective ways to ensure patients are receiving the appropriate level of thickened liquids.

## Summary

- Visual estimation of thickness level in fluids via video is highly inaccurate across practitioners and students, showing that years of professional clinical experience were not associated with improved visual reliability.
- Visual judgement could be biased by product composition under experimental conditions; thickness is more easily recognised in opaque fluids compared to clear ones.
- Relying solely on visual checks may compromise patient safety and increase risks of accidental over‐thickening, leading to behavioural dehydration from reduced fluid intake.
- Multidisciplinary teams and training programmes should not rely on visual judgement but adopt objective screening tools like the IDDSI syringe flow test.

## 1 Introduction

Modifying the consistency of liquids using thickening agents is a primary management strategy applied for patients with oropharyngeal dysphagia [1]. While speech language pathologists (SLPs) typically prescribe the target thickness, the actual preparation and administration of these fluids rely on a multidisciplinary team as well, including clinical dietitians, nursing staff and foodservice workers. As the flow rate of a liquid decreases with increased viscosity, patients with deficits in the temporal coordination of swallowing are afforded more time to safely manipulate and consume the bolus [2, 3]. The immediate physiological benefits of thickened liquids, such as eliminating or reducing instances of aspiration, have been consistently demonstrated under instrumental assessments like videofluoroscopy [4, 5]. However, the long‐term clinical benefits are less direct. The accuracy of fluid preparation is critical; while under‐thickened liquids pose an immediate aspiration risk, over‐thickened liquids carry severe nutritional consequences, including early satiety and altered medication absorption. Consequently, incidences of dehydration have been reported to be greater in patients on thickened liquids as the intake of liquid is likely to be reduced [6, 7], while the occurrence of chest complications, such as pneumonia, has not been proven to be significantly reduced [6, 8]. Although the benefits are not absolute, thickened liquids remain a staple prescription in dysphagia management [1, 9].

To standardise terminology and practices globally, the International Dysphagia Diet Standardisation Initiative (IDDSI) was developed [10]. Thickened liquids are classified into four levels: Level 1 (Slightly Thick), Level 2 (Mildly Thick), Level 3 (Moderately Thick) and Level 4 (Extremely Thick) [10]. The framework introduced simple, objective tools for clinical use. The flow rate of Levels 1–3 can be assessed using the syringe flow test, where the liquid flows freely under gravity from a 10 mL syringe for 10 s; the residual volume classifies the thickness [10]. Extremely thick liquid (Level 4) is assessed by a spoon tilt test to evaluate its adhesiveness and cohesiveness [10]. Although the IDDSI flow rate test is accessible and removes the need for expensive rheological instruments, clinicians, patients and caregivers frequently bypass it in daily practice. Instead, they commonly rely on visual inspection as the liquid is stirred or poured to determine whether it matches the prescribed recommendation [11]. While visual inspection of Level 1–3 liquids is explicitly not recommended by the IDDSI framework, it remains deeply rooted in clinical practice and day‐to‐day beverage preparation.

The reliability of subjective visual assessment is highly questionable. Decades ago, Glassburn and Deem [12] demonstrated that SLPs exhibited immense variability and inconsistency when manually mixing thickeners to target consistencies. Crucially, their foundational research revealed that an SLP's years of clinical experience did not correlate with their ability to accurately produce or judge the target viscosity [12]. Despite this long‐standing evidence highlighting the unreliability of human estimation in fluid mechanics, the practice of visually inspecting liquids persists unchecked across various healthcare settings and professionals; this could be secondary to a lack of standard institutional measurement guidelines or tools at the point of care [13]. The accuracy of preparing thickened liquids is vital to the efficacy of this compensatory strategy, as the swallowing physiology changes with different levels of thickness [14]. Efficacy studies are based on specific, controlled thickening levels. If the liquid consistency used during a clinical or instrumental swallow evaluation differs from what is eventually mixed and consumed by the patient on the ward or at home, the discrepancy could entirely negate the compensatory effect [15], placing the patient at an elevated risk of penetration/aspiration and pharyngeal residue.

Therefore, this study aims to comprehensively evaluate the reliability of visual inspection of thickened liquids. The secondary aim is to determine whether years of clinical experience, practitioner status, or the type of thickening agent used affects this reliability.

## 2 Methods

The study was approved by the Chinese University of Hong Kong Survey and Behavioral Research Ethics Committee (SBRE‐25‐0742).

### 2.1 Stimuli Preparation

Four cups containing 200 mL of thickened liquid (water) were prepared at room temperature. Two different commercially available thickeners were utilised to evaluate differences between clear gums [2] and opaque starches [3]:

1. Level 2 (Mildly Thick) prepared with 2 scoops (2.4 g) of xanthan gum‐based ThickenUp Clear (Nestlé Health Science, Switzerland).
2. Level 4 (Extremely Thick) prepared with 6 scoops (7.2 g) of xanthan gum‐based ThickenUp Clear.
3. Level 1 (Slightly Thick) prepared with 2 scoops (7.6 g) of starch‐based ThickenUp (Nestlé Health Science, Switzerland).
4. Level 4 (Extremely Thick) prepared with 3.5 scoops (13.3 g) of starch‐based ThickenUp.

The liquids were prepared strictly according to the manufacturer's recommendations. The liquids underwent the IDDSI flow test and were confirmed to be at the specific IDDSI level before the video shooting. Videos were recorded of each thickened liquid being picked up by a spoon and then dripping down from the plastic spoon into a cup, mimicking the typical visual inspection method utilised in clinical settings. Each video was at least 12 s in duration. Providing two different IDDSI thickness levels, but not all four, across two types of thickening agents prevented participants from utilising relative comparison to deduce the amounts.

### 2.2 Participants and Procedure

An online survey was distributed to local practising SLPs through professional networks and to final‐year SLP students in a postgraduate programme who have completed two clinical practicums on dysphagia management. The students attended a course on dysphagia and a class on texture modification, in which the concept of the IDDSI framework and testing methods (flow test) were taught. The brand used in this study, Nestle, was also introduced in the course, and it was the brand with the dominant market share locally. In the clinical practicums, they encountered patients with dysphagia, but the exposure and setting varied among students. For local practitioners, they are likely to be familiar with the IDDSI framework and the testing methods, as IDDSI has been introduced in the region for at least 4–5 years prior to this study, but the adoption of IDDSI varies among clinical settings. Similar to students, they should be familiar with the brand and the two types of thickeners, as it has a high market share.

The survey contained demographic questions regarding work setting, years of clinical experience, thickener familiarity and for students, their clinical education experiences. Participants were subsequently presented with the four videos, and they were provided with the brand and type of the thickener that was used to prepare the thickened liquid. Participants were permitted to replay the videos as many times as they preferred. For each video, participants were asked to select the appropriate answer among five choices: The first four choices were in the format of number of scoops (IDDSI level 1/2/3/4) according to the manufacturer's formulae, and a fifth choice of ‘Others’ for participants to freely enter the estimated amount of thickener (in product‐specific scoops) per 200 mL liquid.

### 2.3 Statistical Analysis

Data were analysed using SPSS Statistics (Ver 28.0; IBM, Armonk, USA). Overall accuracy scores (out of 4) were calculated for each participant. Because the scores were ordinal and non‐normally distributed, non‐parametric tests were employed. The Mann–Whitney *U* Test was utilised to compare the overall scores between students and practitioners. To assess the impact of clinical experience, students were assigned ‘0 years’ of experience, merged with the practitioner cohort, and analysed using the Kruskal–Wallis test and Spearman's rank‐order correlation. Chi‐square tests of independence were conducted to compare item‐level accuracy (by video) between the cohorts. To evaluate whether visual estimation accuracy generalised across different thickening agents or reflected a product‐specific bias, a McNemar's test was used to analyse proportional changes in accuracy between the matching extreme thickness conditions: Xanthan gum‐based IDDSI Level 4 and starch‐based IDDSI Level 4. In addition, a Cohen's Kappa (ϰ) coefficient was calculated to assess the strength of inter‐trial agreement between these two thickener bases. A significance level of *p* = 0.05 was adopted.

## 3 Results

### 3.1 Participant Demographics

A total of 107 participants completed the study, comprising 57 practising SLPs and 50 SLP students. Among the practitioners, 49.1% (28/57) worked in residential care homes or hostels, 38.6% (22/57) worked in hospitals, 5.3% (3/57) in private clinics, and 7.0% in other settings such as special schools. Practitioner experience was diverse: 38.6% had ≤ 2 years, 24.6% had 2–5 years, 21.1% had 5–10 years and 15.8% possessed > 10 years of clinical experience. The majority of practitioners (78.9%) utilised both starch and xanthan gum‐based thickeners in their daily work. Among the students, 100% (50/50) reported having prepared thickened liquids during their clinical practicums, and 78% utilised both starch and xanthan gum‐based thickeners.

### 3.2 Overall Accuracy and the Role of Experience

The overall accuracy of visual inspection was calculated across the entire sample. Students correctly identified the thickness in 49.5% (99/200) of trials, while practitioners were correct in 47.4% (108/228) of trials. A Mann–Whitney *U* test confirmed that there was no statistically significant difference in the overall accuracy between students and practising clinicians (*U* = 1502.5, *p* = 0.60). The cohorts were combined to create an experience continuum ranging from 0 years to > 10 years. A Kruskal–Wallis *H* test confirmed that years of clinical experience did not significantly affect the reliability of visual inspection (*H* = 6.56, *p* = 0.16). A Spearman's correlation further confirmed the absence of any linear trend between experience and accuracy (*ρ* = −0.084, *p* = 0.38).

### 3.3 Accuracy by Thickener Type and Level

When evaluating the entire combined cohort (*N* = 107) across individual video trials, considerable performance variance emerged (Table 1). For the xanthan gum‐based samples, the Level 2 (Mildly Thick) liquid was correctly identified by 52.3% (56/107) of participants, whereas the Level 4 (Extremely Thick) clear gum liquid had an accuracy of only 16.8% (18/107). For starch‐based samples, Level 1 (Slightly Thick) was accurately identified by 46.7% (50/107) of all participants, while Level 4 (Extremely Thick) had a combined accuracy rate of 77.6% (83/107). When comparing performance metrics between the two distinct cohorts (Table 1), Level 2 xanthan gum trial did not show any statistically significant difference (*p* = 0.795) between students and practitioners. For Level 4 xanthan gum samples, students were significantly worse than practitioners (*p* < 0.001). For starch‐based samples, students were significantly better than practitioners for both Level 1 (*p* = 0.046) and Level 4 samples (*p* = 0.028).

**Table 1:** Visual inspection accuracy and cohort comparison by video trial.

Base	Target answer	Student accuracy (*n* = 50)	Practitioner accuracy (*n* = 57)	Chi‐square	*p* value
Xanthan gum	Level 2	25 (50.0%)	31 (54.4%)	0.067	0.795
	Level 4	1 (2.0%)	17 (29.8%)	12.816	**< 0.001**
Starch	Level 1	29 (58.0%)	21 (36.8%)	3.977	**0.046**
	Level 4	44 (88.0%)	39 (68.4%)	4.797	**0.028**

*Note:* Bold values indicate statistically significant at *p* < 0.05.

To determine whether visual estimation accuracy generalised across different thickening agents under the experimental condition, a McNemar's test and a Cohen's Kappa coefficient were executed to evaluate the inter‐trial performance agreement between the Level 4 starch and Level 4 xanthan gum conditions. The analysis revealed a profound, highly significant proportional asymmetry between thickener types (McNemar's $\chi^{2}=50.57$‬, *p* < 0.001), paired with a negative statistical agreement metric (ϰ = −0.1085). This result indicated that accuracy is bound to the type of thickener under video‐based assessment conditions.‬‬‬‬‬‬‬‬‬‬‬‬‬‬‬‬‬‬‬‬‬‬‬‬‬‬‬‬‬‬‬‬‬‬‬‬‬‬‬‬‬‬‬‬‬‬‬‬‬‬‬‬‬‬‬‬‬‬‬‬

### 3.4 Teaching and Adoption of Visual Inspection of Thickened Liquid in Students

The survey also asked students about teaching regarding visual inspection during clinical practicums. From the results, 84.0% (42/50) of students reported being explicitly asked by their clinical educators to visually inspect liquid thickness, but 50% (25/50) reported they were never taught how to perform this inspection during the practicum. Despite their low overall accuracy (49.5%), students perceived visual inspection to be a viable clinical tool after their clinical practicums. When asked to rate the perceived accuracy on a scale of 0–5, 70% of students rated it as moderately accurate (3 out of 5 stars). A Spearman correlation revealed a lack of correlation between a student's perceived accuracy of the method and their actual total score (*r* = −0.076).

## 4 Discussion

The primary finding of this study is that visual estimation of the thickness of the liquid categorised by IDDSI level under video‐observed conditions is highly inconsistent, regardless of the thickening agent. With an overall accuracy rate remaining below 50% across cohorts, these video‐based findings suggest that unverified visual judgement is variable and should be supported by objective criteria. The secondary research aim confirmed that visual inspection does not become more accurate with clinical experience. Across all levels of thickened liquid, time spent practising in the profession did not significantly affect accuracy. These findings strongly corroborate the foundational work of Glassburn and Deem [12], who demonstrated decades ago that SLPs were highly inconsistent in manually preparing thickeners and that clinical experience offered no protective advantage against this variability. Some clinicians might expect that repeated exposure to thickened liquids in daily practice would hone their visual judgement. This study indicated that years of clinical practice alone were not associated with improved accuracy in this cohort. In fact, extended experience might render clinicians more prone to systemic error, as entrenched, unverified practices become increasingly difficult to overturn.

### 4.1 Xanthan Gum and Starch Based Thickened Liquid

Further analysis of the error patterns revealed a difference in the performance of visual inspection of thickened levels of liquids and among respondents. Students significantly outperformed practitioners in identifying Level 4 starch liquids (88% vs. 68%), but failed almost entirely (2.0% accuracy) to identify Level 4 xanthan gum liquids. This sharp performance divergence highlights a possible product‐specific perceptual bias rather than a generalised failure of vision under the current experimental conditions. Starch‐based thickeners inherently present highly visible cues, becoming cloudy, opaque and visibly granular at higher concentrations [14]. When managing xanthan gum thickeners, which are engineered to retain clear, translucent uniformity across all viscosity levels [2], it might be more difficult to discriminate levels of thickness. Practitioners and students appear to rely on this visual cloudiness as a learned heuristic for thickness under these video‐based assessment conditions, especially the more extreme end of thickened liquid. However, the current study did not have all levels of thickened liquid between the two types of thickeners for a more valid comparison.

The clinical implications of this could be significant. If a patient requires Level 4 xanthan‐gum‐based liquids to prevent aspiration, but a clinician, nurse, or patient assistant relies on visual inspection and assumes it is only Level 2 because it lacks cloudiness, they will continuously add thickener. This systemic error pattern means patients evaluated via visual inspection will likely consume liquids that are significantly thicker than the consistency used during their instrumental evaluation. Consuming excessively thick liquids that are unsuitable for the patient's specific swallowing mechanism drastically increases the risk of pharyngeal residue, post‐swallow aspiration and dehydration [6].

### 4.2 Eradicating the Hidden Curriculum

The study also uncovers a potential problem within clinical education. An overwhelming 84% of students are instructed by their clinical educators to perform visual inspections during practicums, despite 50% receiving no formal instruction on the practice. Compounding this issue is the finding that students did not have an accurate insight into their own inaccuracy under video‐based conditions (*r* = −0.076). Clinical educators could have inadvertently reinforced a scientifically unsupported, dangerous practice. By asking students to visually inspect the thickness in liquids, they legitimise visual inspection as a valid clinical tool, setting up the next generation of SLPs to perpetuate the exact inconsistencies first identified by Glassburn and Deem [12]. While this study evaluated SLPs, subjective visual inspection could be pervasive across nursing, dietetic and foodservice training programmes as well. The multidisciplinary nature of dysphagia management means that clinical educators across all allied health fields should collaborate to correct this. To ensure patient safety, educational programmes across all disciplines should advocate the use of objective methods, such as the IDDSI syringe flow rate test or adhering to the formula for the corresponding level.

### 4.3 Limitations

There were limitations to this study. The study only included speech‐language pathologists and students from one geographical region; the problem might be confined to a specific region and group of SLPs. Further multi‐site studies should be conducted to determine if this was a phenomenon across the globe. Furthermore, evaluating fluids via an online video format limits ecological validity. In live clinical environments, healthcare staff utilise a multimodal sensory approach, evaluating pouring dynamics, tactile spoon coating, cup tilt and the physical resistance experienced during manual stirring. Because our video‐based design isolated purely visual fluid flow and removed tactile‐kinetic feedback, it may underestimate real‐world accuracy. This limits the generalisation of the findings from this experimental study; future research must evaluate accuracy using live, hands‐on mixing methods, or even the reliability of thickened liquid preparation and how it affects clinical outcomes. The last limitation is that, due to the anonymous and online nature of the survey, intra‐rater reliability testing could not be conducted. Future studies should adopt study designs that could capture intra‐rater reliability measurements.

## 5 Conclusion

Fluid modification using thickening agents is a vital compensatory strategy in dysphagia management. Its efficacy and safety depend on the precision of the thickened liquid used during both assessment and daily consumption. To maximise patient safety and nutritional optimisation, the integration of objective tools, such as the IDDSI syringe flow rate test, should be encouraged as a supplementary quality‐control standard, rather than relying solely on individual visual estimates. Clinicians, educators, patients and caregivers should be encouraged to adhere to objective manufacturer ratios or routinely utilise the IDDSI syringe flow rate test [10].

## Author Contributions

**Raymond Fong:** conceptualisation, data collection and analysis, and writing the manuscript. **Connie C. Y. Kwan:** data analysis, writing the manuscript, and review.

## Funding

The authors have nothing to report.

## Ethics Statement

The study was approved by the Chinese University of Hong Kong Survey and Behavioural Research Ethics Committee. The authors declare that the research complied with the Declaration of Helsinki.

## Conflicts of Interest

The authors declare no conflicts of interest.

## Data Availability

The data that support the findings of this study are available from the corresponding author upon reasonable request.

## References

[1] C. M. Steele , W. A. Alsanei , S. Ayanikalath , et al., “The Influence of Food Texture and Liquid Consistency Modification on Swallowing Physiology and Function: A Systematic Review,” Dysphagia 30, no. 1 (2015): 2–26. 25343878 10.1007/s00455-014-9578-x PMC4342510

[2] E. K. Hadde , B. Mossel , J. Chen , and S. Prakash , “The Safety and Efficacy of Xanthan Gum‐Based Thickeners and Their Effect in Modifying Bolus Rheology in the Therapeutic Medical Management of Dysphagia,” Food Hydrocolloids for Health 1 (2021): 100038.

[3] R. J. Dewar and M. J. Joyce , “Time‐Dependent Rheology of Starch Thickeners and the Clinical Implications for Dysphagia Therapy,” Dysphagia 21, no. 4 (2007): 264–269. 10.1007/s00455-006-9050-7 17216389

[4] J. Robbins , G. Gensler , J. Hind , et al., “Comparison of 2 Interventions for Liquid Aspiration on Pneumonia Incidence: A Randomized Trial,” Annals of Internal Medicine 148, no. 7 (2008): 509–518. 18378947 10.7326/0003-4819-148-7-200804010-00007 PMC2364726

[5] J. A. Logemann , G. Gensler , J. Robbins , et al., “A Randomized Study of Three Interventions for Aspiration of Thin Liquids in Patients With Dementia or Parkinson's Disease,” Journal of Speech, Language, and Hearing Research 51, no. 1 (2008): 173–183. 10.1044/1092-4388(2008/013) PMC2894528 18230864

[6] S. Werden Abrams , P. Gandhi , and A. Namasivayam‐MacDonald , “The Adverse Effects and Events of Thickened Liquid Use in Adults: A Systematic Review,” American Journal of Speech‐Language Pathology 32, no. 5 (2023): 2331–2350. 37437527 10.1044/2023_AJSLP-22-00380

[7] A. P. Vivanti , K. L. Campbell , M. S. Suter , M. T. Hannan‐Jones , and J. A. Hulcombe , “Contribution of Thickened Drinks, Food and Enteral and Parenteral Fluids to Fluid Intake in Hospitalised Patients With Dysphagia,” Journal of Human Nutrition and Dietetics 22, no. 2 (2009): 148–155. 19302120 10.1111/j.1365-277X.2009.00944.x

[8] A. Kaneoka , J. M. Pisegna , H. Saito , et al., “A Systematic Review and Meta‐Analysis of Pneumonia Associated With Thin Liquid vs. Thickened Liquid Intake in Patients Who Aspirate,” Clinical Rehabilitation 31, no. 8 (2017): 1116–1125. 28730887 10.1177/0269215516677739

[9] J. M. Garcia , E. Chambers , and M. Molander , “Thickened Liquids: Practice Patterns of Speech‐Language Pathologists,” American Journal of Speech‐Language Pathology 14, no. 1 (2005): 4–13. 15962843 10.1044/1058-0360(2005/003)

[10] J. A. Y. Cichero , P. Lam , C. M. Steele , et al., “Development of International Terminology and Definitions for Texture‐Modified Foods and Thickened Fluids Used in Dysphagia Management: The IDDSI Framework,” Dysphagia 32, no. 2 (2017): 293–314. 27913916 10.1007/s00455-016-9758-y PMC5380696

[11] C. E. A. Barbon and C. M. Steele , “Thickened Liquids for Dysphagia Management: A Current Review of the Measurement of Liquid Flow,” Current Physical Medicine and Rehabilitation Reports 6, no. 4 (2018): 220–226. 32149018 10.1007/s40141-018-0197-6 PMC7059648

[12] D. L. Glassburn and J. F. Deem , “Thickener Viscosity in Dysphagia Management: Variability Among Speech‐Language Pathologists,” Dysphagia 13, no. 4 (1998): 218–222. 9716753 10.1007/PL00009575

[13] X. S. Wu , A. Miles , and A. Braakhuis , “The Effectiveness of International Dysphagia Diet Standardization Initiative‐Tailored Interventions on Staff Knowledge and Texture‐Modified Diet Compliance in Aged Care Facilities: A Pre‐Post Study,” Current Developments in Nutrition 6, no. 4 (2022): nzac032. 35415388 10.1093/cdn/nzac032 PMC8994209

[14] C. M. Steele , M. Peladeau‐Pigeon , C. A. E. Barbon , et al., “Reference Values for Healthy Swallowing Across the Range From Thin to Extremely Thick Liquids,” Journal of Speech, Language, and Hearing Research 62, no. 5 (2019): 1338–1363. 10.1044/2019_JSLHR-S-18-0448 PMC6808317 31021676

[15] R. Goulding and A. M. Bakheit , “Ealuation of the Benefits of Monitoring Fluid Thickness in the Dietary Management of Dysphagic Stroke Patients,” Clinical Rehabilitation 14, no. 2 (2000): 119–124. 10763787 10.1191/026921500667340586

